# A psychological autopsy study of suicide among Inuit in Nunavut: methodological and ethical considerations, feasibility and acceptability

**DOI:** 10.3402/ijch.v72i0.20078

**Published:** 2013-03-26

**Authors:** Eduardo Chachamovich, Jack Haggarty, Margaret Cargo, Jack Hicks, Laurence J. Kirmayer, Gustavo Turecki

**Affiliations:** 1 Department of Psychiatry, McGill Group for Suicide Studies, McGill University, Montreal, Quebec, Canada; 2 Faculty of Medicine, Northern Ontario School of Medicine, Sudbury, Ontario, Canada; 3 School of Health Sciences, University of South Australia, Mawson Lakes, South Australia, Australia; 4 University of Greenland, Nuuk, Greenland; 5 Division of Social and Transcultural Psychiatry, Department of Psychiatry, McGill University, Montreal, Quebec, Canada

**Keywords:** suicide, Inuit, psychological autopsy, mental health, cross-cultural, Canada, Aboriginal, Nunavut, risk factors

## Abstract

**Introduction:**

The increasing global prevalence of suicide has made it a major public health concern. Research designed to retrospectively study suicide cases is now being conducted in populations around the world. This field of research is especially crucial in Aboriginal populations, as they often have higher suicide rates than the rest of the country.

**Objective:**

This article presents the methodological aspects of the first psychological autopsy study on suicide among Inuit in Nunavut. *Qaujivallianiq Inuusirijauvalauqtunik* (*Learning from lives that have been lived*) is a large case-control study, including all 120 cases of suicide by Inuit that occurred in Nunavut between 1 January 2003 and 31 December 2006. The article describes the research design, ethical considerations and strategies used to adapt the psychological autopsy method to Nunavut Inuit. Specifically, we present local social and cultural issues; data collection procedures; and the acceptability, reliability and validity of the method.

**Method:**

A retrospective case-control study using the psychological autopsy approach was carried out in 22 communities in Nunavut. A total of 498 individuals were directly interviewed, and medical and correctional charts were also reviewed.

**Results:**

The psychological autopsy method was well received by participants as they appreciated the opportunity to discuss the loss of a family member or friend by suicide. During interviews, informants readily identified symptoms of psychiatric disorders, although culture-specific rather than clinical explanations were sometimes provided. Results suggest that the psychological autopsy method can be effectively used in Inuit populations.

Suicide, especially among youth, is one of the most important causes of death worldwide (1). Due to its increasing prevalence, suicide is now considered a major public health concern (2). In countries including Canada, New Zealand and Australia, indigenous peoples have much higher suicide rates than the rest of the population (3). In 2000, Canadian Aboriginal suicide rates were 24 per 100,000 (2 times the general Canadian rate). Among Aboriginal populations in Canada, Inuit have among the highest rates. Between 1999 and 2003, their rates were 135 per 100,000 (4, 5). Historically, Inuit have had relatively low suicide rates. However, in the past 20 years, they have more than tripled (6). This drastic increase in suicides is almost entirely due to an exponential increase in suicides by individuals younger than 25 years old (7).

From 2005 to 2010, our group conducted large case-control study on suicide among Nunavut Inuit called *Qaujivallianiq Inuusirijauvalauqtunik* (*“Learning from lives that have been lived”*) which included all 120 cases of suicide by Inuit that occurred in Nunavut between 1 January 2003 and 31 December 2006. This article describes the strategies used to adapt the psychological autopsy method to the investigation of suicide among Inuit living in Nunavut and presents the research methodology in detail. Findings from the study will be reported in subsequent publications.

## Studying the origins of suicide

Suicide is a multidimensional and complex phenomenon resulting from the interaction of many factors (8). In the case of suicide among Inuit, social and cultural changes, poverty, geographical isolation, cultural suppression and political disempowerment may all play a role in the rapid increase in suicide rates (9–11). Acculturation, which denotes changes to one culture when it comes in contact with another, is also frequently implied as a causal factor for suicide (4, 12). The association between greater Inuit contact with southern Euro-Canadian culture and increased rates of suicide is still disputed, due to the inconsistent findings across Inuit groups (13, 14). What seems indisputable, however, is that substance misuse and mood disorders, play a key role in the recent increase of suicide rates among Inuit (4, 15–19).

The current international literature is marked by a mismatch between the enormous impact of suicide on Inuit communities and the paucity of scientific investigations in this field (7, 20). There are some studies on suicide attempts among Inuit but few on people who have died by suicide. Those who attempt and those who commit suicide may differ in important ways.

This gap in the literature may be due, in part, to the methodological challenges involved in conducting studies on death by suicide compared to suicide attempts. In most populations, death by suicide is a rare event, and it is therefore not feasible to conduct prospective studies. Retrospective case-control designs using the psychological autopsy method to compare individuals who died by suicide with matched controls are practical alternatives to prospective studies.

## The psychological autopsy method

The psychological autopsy method was originally developed by Shneidman (21) as a tool to determine the cause of a suspicious death (i.e. to differentiate suicides from killings) in forensic examinations (21, 22). Soon after its introduction, it was used in scientific investigations by researchers interested in the causes of suicidality (22–24). The psychological autopsy or retrospective reconstruction method is a widely used approach to study suicide in many different populations with good performance, including Europe (25–27), North-America (28–32), China (33–35), South-America (36) and Taiwanese Aboriginal groups (14, 37). The objective of a psychological autopsy is to gather information about the circumstances surrounding an individual's death so as to understand the reasons behind the suicide (22). The method consists of interviewing close relatives and/or friends of the deceased using a combination of open-ended questions and standardised instruments that can generate psychiatric diagnoses, as well as identify developmental or environmental risk and protective factors that may have been associated with the suicide (Table I).

Information is usually obtained from multiple sources, including those closest to the deceased (22, 26). For personal and developmental history, a parent or a partner is usually the most appropriate informant, when available. In the case of young suicides, a sibling or a close friend may also be important to provide information about aspects of the individual's life that parents may have been unaware of, such as drug use or interpersonal problems (22, 24). Obtaining information from health service providers can offer details about medical treatments, use of medications, and other mental and physical problems that may not be adequately remembered by other informants. Whenever possible, interviewing more than 1 informant independently is recommended (22). Similarly, interviewing informants who are related to the individual in different ways may allow access to complementary information. The timing of the interview is also important; the informant should not be approached too soon after the death (to avoid triggering an intense emotional reaction), but not too late either (to avoid recall bias).

The interviews used in psychological autopsies are semi-structured. They combine open-ended questions with standardised instruments. Open-ended questions are suitable to collect information about the trajectory of life events, childhood development and parent–child relationships. Standardised instruments can gather information about psychiatric disorders, impulsiveness, aggression, childhood abuse and personality traits. The selection of instruments for psychological autopsy interviews is dependent on the objectives of each study (36). Typically, assessments of DSM-IV Axis I psychiatric disorders and personality disorders/traits are included, due to the consistently demonstrated association between psychopathology and suicide (38–40). The instruments used most frequently to assess diagnoses are the SCID I and II (41).

**Table I:** Suicide-related factors that were investigated with psychological autopsy interviews

Circumstances of death (method, motivation, intoxication of alcohol/drugs)
Family history of suicide behaviour and/or psychopathology
Experiences of childhood and adolescence
Interpersonal relationship: parents, siblings, partner, children, friends
Social support and isolation
Housing
Legal problems
Impulsive-aggressive behaviours
Psychiatric history and current diagnoses
Personality disorders and trait

Psychopathology alone does not account for suicide. For example, while major depression is the most prevalent diagnosis among those who die by suicide, the vast majority of people with major depression do not make suicide attempts (8, 42, 43). Other factors that independently contribute to suicide completion among individuals with major depressive disorder include impulsiveness, aggressiveness and exposure to childhood abuse (8, 43–48). Thus, the inclusion of measures to assess these factors is important in psychological autopsy studies.

## Reliability and validity of psychological autopsies

Several elegant studies have demonstrated the validity and reliability of the psychological autopsy method. Conner and colleagues (29, 30) showed that proxy-based data have substantial diagnostic agreement when compared to data gathered from individuals themselves regarding suicide attempts (percentage of agreement varied from 81 to 100%). In the same sample, they were able to show substantial agreement between proxy-based data and first-hand reports regarding observable events of life. Schenider et al. (49) also tested the concordance of diagnoses based on information from living controls and informants. They reported *kappa* coefficients above 0.65 for inter-rater and test-retest reliability and above 0.79 for most Axis I disorders (49). Kelly and Mann (28) compared the diagnoses obtained by psychological autopsy against the clinician's ante-mortem diagnosis for 131 subjects. Their findings also support the high reliability of psychological autopsy diagnoses: *kappa* coefficients of 0.84 for Axis I diagnoses and 0.65 for Axis II disorders (28). The *kappa* coefficient is considered a more robust measure than a simple percentage of agreement calculation because it takes into account the agreement occurring by chance.

While psychological autopsy studies have mainly been carried out in Western populations (European and North American), this methodology has been applied more recently to non-Western populations. Zhang et al. (33, 34) demonstrated that the psychological autopsy method had good reliability in a Chinese sample of suicide completers and controls. Werlang and Botega (36) also tested the reliability of a semi-structured interview for the psychological autopsy method in a Brazilian sample and reported high agreement between judges on a series of measures (36). Other studies have successfully used the same methodology in India (50) and Taiwan (37). These positive findings support the high validity and reliability of psychological autopsies for both suicide completion and control groups. In fact, some of these studies were based exclusively on interviews. When taking into account the other sources of information (such as medical charts), we expect the reliability and validity to be even higher.

## Ethical issues

Psychological autopsy studies raise specific ethical issues. Particular care is required when approaching bereaved relatives and friends of those who died by suicide. In the case of Inuit communities, suicide directly affects both the immediate family and friends of the deceased and the entire community (2). Thus, consent to take part in the study should be provided at an individual level, as well as at community and governmental levels.

For this project, we formed a network of collaborators who were familiar with Inuit and other Aboriginal communities and who were well versed in the ethical issues of the study before beginning their fieldwork. In keeping with the principles of participatory research (51) and the Canadian Institutes of Health Research Guideline for Health Research Involving Aboriginal People (52), we sought to involve representatives from Inuit communities and government organisations in the implementation, interpretation and dissemination of the research. The project received crucial input and support from the Government of Nunavut (the Office of the Chief Coroner and the Department of Health and Social Services), the representative Inuit organisation Nunavut Tunngavik Incorporated, the Royal Canadian Mounted Police (RCMP) and the Embrace Life Council (a multisectorial suicide prevention body). In addition, the research project was reviewed and approved by the Institutional Ethics Review Board of McGill University.

In order to minimise emotional distress and avoid the initial period of bereavement, we decided to approach families and friends of the deceased no earlier than 1 year after the death. In addition, the project was explained in great detail to each participant before the interview, and a consent form was provided in both English and Inuktitut. Informants were asked the language in which they would prefer to be interviewed. The choice was often English, especially among younger informants. When an informant requested that the interview take place in Inuktitut, they were invited to select an interpreter (professional or non-professional) with whom they were comfortable speaking openly. Participants were free to stop the interview at any point, as well as to interrupt and resume later on. The interviewers reassessed the participant's will to continue several times during the interview (which lasted typically about 2 hours).

Finally, separate consent forms were collected to review the medical charts of the individuals who died by suicide and the controls. For the former, consent was obtained from the closest first-degree relative (typically one of the parents). The controls provided consent for the review of their own medical records and indicated which informants the researchers should approach. Participants were financially compensated for their contribution (100 Canadian dollars) and could opt to receive the payment or to donate the compensation to an organisation of their choice.

Individuals’ privacy and confidentiality were respected at all times. All names and personal details were omitted in research protocols and reports. As well, the interviews were always conducted in places where the informants’ privacy was assured. Given the high density of inhabitants per household in Nunavut, this was made possible by the personnel from health centres and local community administration.

## The Nunavut suicide case-control study: methodology

Before approaching each community, the research coordinator contacted the nurse in charge of the community health centre to inform them of our project and to collect any relevant information on the current status of the community (i.e. if any recent suicides or other deaths had occurred, if any important events such as feasts or tournaments were planned). Upon arriving in the community, we met with the senior administrative officer and/or the mayor, the nurse in charge, and the RCMP to report our presence in the community and gather additional information. We discussed the families of the cases and controls (potential informants) with the nurse in charge (and sometimes with the mental health nurse, in communities that had one) in order to prepare for issues that could be raised during interviews. For example, it was crucial to ensure that no other deaths (by suicide or otherwise) had occurred in the family after 2006. In addition, it was important that the local professionals and community members be aware of the study in case informants needed assistance of any kind.

**Fig. 1:**
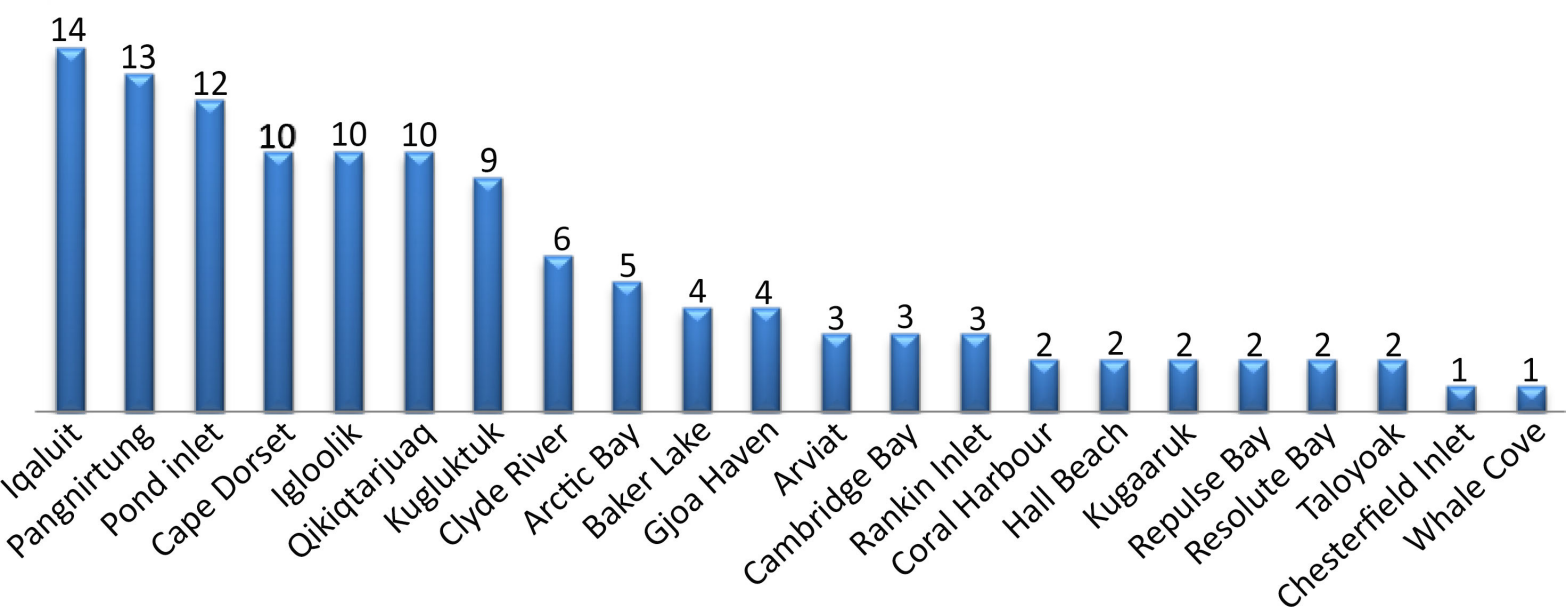
Number of suicides per community in Nunavut between 2003 and 2006.

Our *Qaujivallianiq Inuusirijauvalauqtunik* (*Learning from lives that have been lived*) project included all 120 cases of suicide by Inuit that occurred in Nunavut between 1 January 2003 and 31 December 2006, and 120 controls matched on age, gender and community of origin (Fig. 1). The coroner identified cases of suicide included in our study after performing a physical autopsy, reviewing medical records and gathering information from the RCMP. Controls were selected from the Nunavut Health Care Registration File by identifying individuals who could be matched with the suicide group on community of origin, gender and date of birth.

The mean age of the cases was 23.4 years (SD 9.13). Since the interviews took place after the completion of the suicide (mean Δ=39.6 months, SD 21.06), the controls were older than the deceased at the time of the suicide. We analysed the difference between the dates of birth for each pair of case and control. To do so, we transformed the raw differences in days into absolute values, since controls could be selected even if they were born earlier than the case. As a result, the estimation of the raw differences yielded both positive and negative values that, if not transformed, might produce artificially minimal means and standard deviations. The mean difference between cases’ and control's date of births was 59.27 days (SD 72.26 days), with a minimum difference of 1 day and a maximum of 410 days.

Among the suicide cases, 99 (82.5%) were male and 21 (17.5%) were female. The individuals varied in their method of suicide. Overall, 96 individuals died by hanging (80.0%), 19 by gunshot (15.9%), 4 by stabbing (3.3%) and 1 by overdose (0.8%).

## Description of the psychological autopsy interviews

From March 2006 to July 2010, a total of 498 interviews were carried out with informants in 22 communities across Nunavut. A standardised methodology was used for all the interviews to ensure comparability between cases and controls. For the cases, first-degree relatives (mostly parents) were approached for consent and then interviewed. The living controls were contacted first due to ethical reasons, but were not interviewed. Rather, controls’ relatives and/or friends were interviewed in order to keep the same methodology across both groups.

Overall, 498 interviews were conducted to obtain information on 240 individuals (120 cases and 120 controls). The number of informants per case or control was determined by the interviewer, based on the quality of the interviews and the amount of information gathered. For cases, 279 interviews were conducted (an average of 2.3 interviews per case), whereas for controls 219 interviews were carried out (an average of 1.8 interviews per control). Some interviews were carried out with 2 informants present (typically mother and father of a case). The majority of interviews were carried out with mothers, siblings, partners and friends of cases and controls (Table II).

**Table II:** Types of informants used for the psychological autopsy interviews for both the case and control groups and the frequency that each type of informant was interviewed

Informant	Cases (*N*)	Controls (*N*)
Father	22	17
Mother	57	41
Aunt/uncle	17	9
Sibling	46	52
Grandparent	8	2
Friend	66	46
Partner	41	39
Cousin	12	12
Son/daughter	3	1
Niece/nephew	3	0
High school teachers/guidance counsellors	4	0
Total	279	219

Note: the number of informants is higher than the number of interviews due to the fact that interviews were carried out with more than one informant.

Instruments used in the study measured socio-demographic data, psychopathology, impulsiveness, aggressiveness, history of suicide attempts, family history of psychopathology, development and life trajectory. The selection of these instruments reflected the major risk factors for suicide completion (i.e. previous suicide attempts, psychopathology, familial antecedents, high levels of impulsivity and aggression) (31, 40, 53, 54). Our group was well experienced with these instruments from using them in similar studies in Quebec (Table III).

**Table III:** Description of the instruments that were used in the study

Socio-demographics (information on demographics, alcohol, drugs, physical/sexual abuse, legal problems, medication, education/work)
Life overview (open-ended questions to record informant's subjective perceptions)
SCID I (generates Axis I diagnoses according to DSM-IV)
SCID II (generates Axis II diagnoses according to DSM-IV)
Family antecedents of psychiatric disorders (retrieve information on family psychopathology)
Barratt Impulsivity Scale (BIS-30)
Brown and Goodwin Lifetime History of Aggression (BGLHA)
Suicide History Scale (collect data on previous suicide attempts and ideation)
Life Trajectory Scale (detailed information on childhood, adolescence and adulthood experiences)
Genealogical map (detailed relationship with family members)

The instruments were thoroughly reviewed prior to the fieldwork to ensure that their content was appropriate for the Inuit context. Some items of the Life Trajectory Scale were modified, and others added in order to encompass important aspects of life in Inuit culture, such as experiences with *qallunaat* (white people), pride in Inuit culture, experiences with residential schools, opportunities to hunt and fish, ability to speak English, contact with the government and thoughts for the future of Nunavut among others.

The interviews were followed by a complete review of relevant medical records. For the controls, medical charts were available locally at the community's health centre. The charts for suicide cases in the Qikiqtani region were stored in the Qikiqtani Regional Hospital, while the charts of suicide cases in the Kivalliq and Kitikmeot regions were stored in the health centres of each deceased individual's community. With the available qualitative and quantitative data collected during the interviews and the information from the medical charts, the interviewer was able to determine whether each individual met specific SCID criteria for Axis I and II psychiatric diagnoses. Additionally, with information collected during the interview, the interviewer completed the measures of impulsivity and aggressiveness, history of suicidal behaviour, family antecedents of psychiatric disorders and the individual's life trajectory. Finally, the interviewer wrote a clinical-biographical narrative for each case or control, in which details of the individual's life were summarised. The biographical narrative described the individual's upbringing, familial relationships, academic performance, romantic experiences, interpersonal relationships, occupational life and detailed information about any psychiatric symptoms. This narrative, a copy of the medical records, and the completed set of instruments were sent to the coordinating centre for further processing.

At the coordinating centre, the instruments were assessed to ensure completeness. Any discrepancy in information between instruments (or between an instrument and the content of the narrative summary) was identified and resolved by discussion with the interviewer. The narratives were then blinded to the group each individual was in so that cases and controls could not be obviously distinguished (i.e. the case was disguised, details on the circumstance of death were removed and verbs were all changed to the past tense). The standardised case narrative, a summary of the medical records, and the final Axis I and II diagnoses were then forwarded to a panel of research collaborators to validate the final Axis I and II diagnoses that were given by the interviewer.

**Fig. 2:**
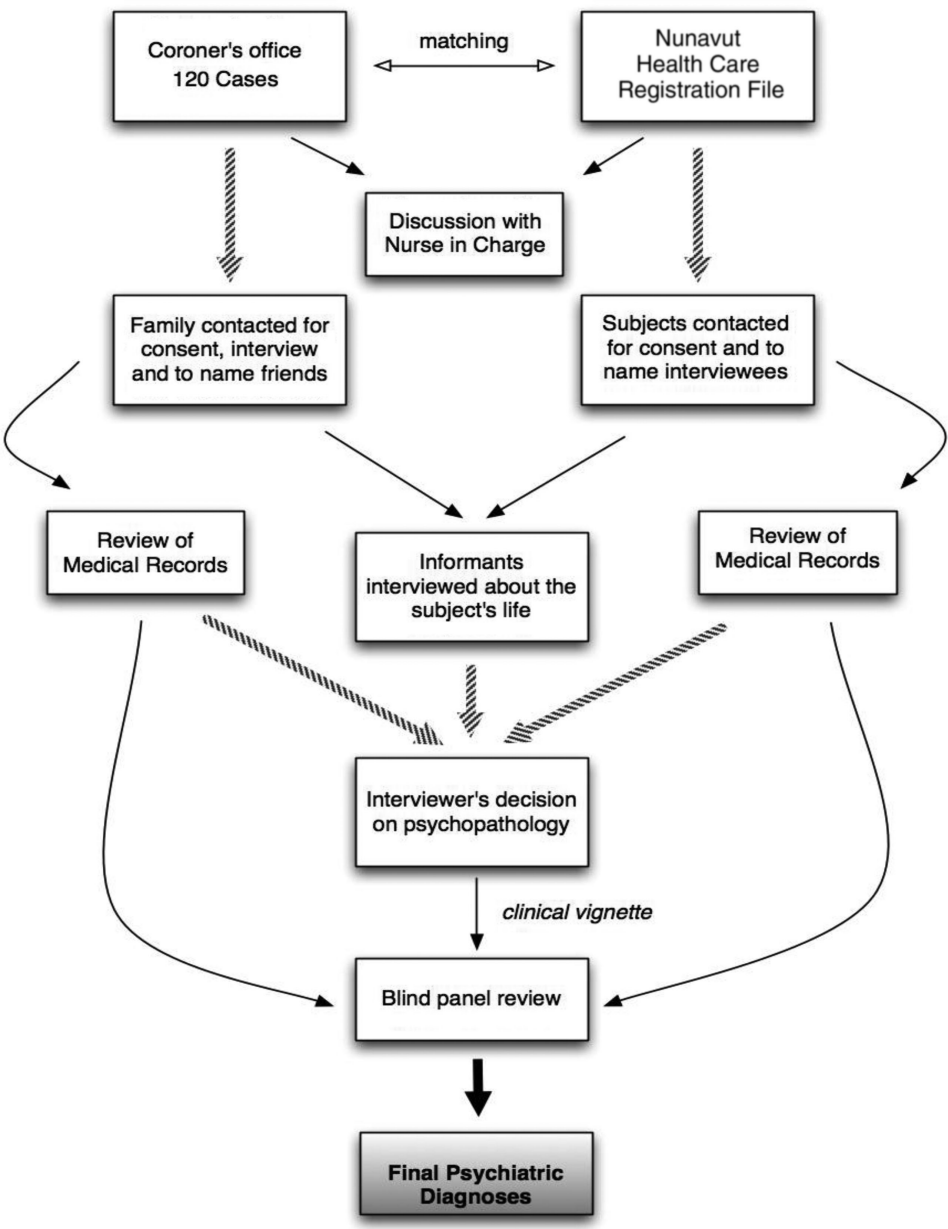
Flowchart of the procedures for data collection and evaluation.

The panel was composed of a clinical psychiatrist, a clinical psychologist with research experience and a senior research coordinator with extensive experience with the SCID I and II instruments. A detailed examination of the diagnostic criteria was carried out for each participant, in order to validate or change the diagnoses. This included determining whether the developmental and clinical history, as described by the narrative, supported the clinical diagnoses. DSM-IV-TR criteria were used as the basis for these assessments. An example of a change made by the panel involved replacing an original diagnosis of adjustment disorder with depressed mood by major depressive episode in a situation where the depressive symptoms started just after an important loss, but included consistent suicidal ideation and gestures, sadness, anhedonia, guilt and motor retardation. Particular attention was also paid to diagnoses of substance abuse (since the informants sometimes minimised levels of substance abuse, even though the medical records and narratives made the level of abuse clear). Finally, the panel carefully verified Axis II criteria to detect potential overlap with Axis I symptoms, such as items for paranoid personality disorder being endorsed in the presence of a schizophrenia diagnosis. Typically, panel sessions lasted 1.5–2 hours, and 7–10 cases were examined per session (Fig. 2).

## Assessing the validity of SCID I and II

We carried out 30 interviews with participants from the control group to assess the validity of SCID I and II interviews. This method determines the level of convergence between self-reports and the informant's reports on our measures. Our team members were blinded to the information collected from the proxy-based interviews and carried out identical interviews with 30 controls, rating SCID I and II diagnoses following the same method that had been applied in all interviews. The information collected by these interviews was used only to estimate the validity of the method and was not incorporated into the controls’ files for the study. We calculated the level of agreement between the DSM-IV Axis I and II diagnoses obtained from the proxy-based interviews (the method of the study) and the interviews with the subjects themselves (the “gold standard” assessment) (55). Overall, we found moderate to high levels of agreement. *Kappa* coefficients were 0.84 for mood disorders, 0.83 for personality disorders, 0.79 for schizophrenia and 0.57 for substance abuse. Among these individuals, 15 had at least 1 Axis I diagnosis (either current or past). Only 7 individuals had 1 or more personality disorder.

## Assessing inter-rater reliability

To assess inter-rater reliability, 15 audiotaped psychological autopsy interviews were selected from the McGill Group for Suicide Studies data bank. The interviews selected were conducted by one of the researchers that carried out the interviews in Nunavut, and listened to and re-rated by another interviewer independently. Both the ratings of the original interviewer and the re-rater were assessed. The *kappa* coefficients demonstrated substantial agreement: 0.82 for mood disorders, 0.79 for substance abuse and 1.0 for schizophrenia.

## Feasibility and acceptability of the psychological autopsy method in Nunavut

Overall, the vast majority of individuals approached agreed to participate in the study. No entire family refused to be interviewed, so interviewers were able to collect information on all cases and controls that were approached. In a few families, 1 or 2 members declined to participate because they did not feel comfortable talking about the suicide. Nevertheless, these family members did not oppose the participation of other family members.

Only 1 interview out of 498 had to be interrupted because of emotional distress, and therefore only 1 interview remained incomplete. Although several interviews were highly emotional and discussed intensely personal issues, most informants did not have negative reactions. On the contrary, participants of the case group (i.e. relatives or friends of the deceased) often voiced appreciation for the opportunity to talk about their feelings, as they had not received much attention following the suicide. Typically, reactions among the relatives and friends of the control group were neutral. This high level of acceptability for both groups is reassuring. However, it is important to recall that whenever possible the nurse in charge was contacted at the local community health centre prior to the interviews, and so certain potential informants were not contacted based on the nurse in charge's advice. The goal was to identify informants that knew the participant well and would be comfortable answering questions about him or her.

The researchers found a high degree of face validity in the instruments used. Some items, however, may have been confounded due to the social context. For instance, the Barratt Impulsivity Scale (56) items number 16 (“I change jobs”) and 21 (“I change residences”) may provide misinterpreted or limited information due to the important lack of housing and employment in Nunavut (57). For example, 75.2% of controls and 82.1% of cases responded “rarely” or “never” for those items, respectively. However, both participants who rarely or never changed jobs and houses and those who had no job or house would have submitted the same answers. Therefore, these answers were not adequate to assess low and high impulsivity in Inuit populations.

The literature in cultural psychiatry has raised questions about the cross-cultural applicability of conventional diagnostic categories and criteria. In this context, it is significant to note that the informants were able to understand questions related to symptomatology of the Axis I disorders. Particularly core symptoms of major psychiatric syndromes (such as major depression or psychotic conditions) were promptly recognised, and the participants were able to give clear examples. Often, the content of the interviews was confirmed by content of the medical charts. Informants were consistently able to recognise the expression of psychiatric symptoms (e.g. auditory hallucinations, depressed mood, lack of interest or pleasure in the usual activities and social isolation). Explanations for the occurrence of symptoms, however, were often based on the Inuit cultural background, but this did not prevent recognition of the symptoms as pathological. For example, auditory hallucinations were described in 2 interviews as the revenge of an animal spirit that had been maltreated (according to Inuit tradition) by an Inuit hunter. Nevertheless, informants could identify the symptom as unhealthy and could indicate the point in time that it emerged. Again, these details were corroborated with medical charts. Additionally, unlike what is frequently the case in southern Canadian samples, Inuit informants were remarkably open and direct when describing alcohol and drug use (although they often underestimate their levels of misuse) as well as family violence and abuse. Thus, although the interpretation of a symptom or behaviour might differ from that of Western psychiatry, there was no difficulty in collecting information on signs and symptoms of psychopathology.

The majority of informants had positive responses to the interviews. Frequently, informants thanked the researchers for the opportunity to voice their feelings of grief caused by the suicide of a loved one. They mentioned that it was an important part of their healing process. In addition, the fact that the interviewers were “outsiders” was generally viewed as a positive aspect of the study. Since most communities in Nunavut have both a small number of inhabitants and complex networks of relationships, it can be challenging for some people to disclose intimate information. The interview gave people an opportunity to speak openly without fearing any negative repercussions within their family or community.

## Discussion and conclusion

This article describes the adaptation and implementation of the psychological autopsy method to investigate suicide among Inuit in Nunavut. This is the first study in Nunavut to explore suicide with this method. Our initial findings suggest that the psychological autopsy method, as described in this article, was feasible, well accepted and valid. Informants easily understood questions in the interviews and could answer them with detail and accuracy. There were very high rates of participation and minimal instances of interruption during interviews. Interviewers indicated that the informants were comfortable taking part in the interviews, and many informants were openly appreciative of the opportunity to speak about their lost family member or friend.

Informants appreciated that interviews were private and confidential. They formed positive a relationship with the interviewer and were reassured by the fact that the interviewer was an “outsider” who did not know people in the community. Some also expressed that the interview helped individuals come to terms with the death and the challenges faced by the deceased before they passed away.

Previous studies have reported the therapeutic effect of psychological autopsies (22, 58–60). Factors such as experiencing connectedness, obtaining psychological support and accepting the loss may contribute to the positive impact of the psychological autopsy approach. Our findings support the positive effects of this methodology among Nunavut Inuit.

The need for research on suicide among Inuit was recognised by the vast majority of informants as well as by health professionals in Nunavut. Overall, the attitude towards the project was positive. Authors have consistently identified the urgent need for effective interventions (61–63), and Inuit individuals also pointed out this need during interviews. Respondents hoped that the study might contribute to suicide prevention in future Inuit generations.

As an evaluation of the feasibility and acceptability of the psychological autopsy method in a specific cultural context, the present study has important limitations. There was no standardised assessment to measure the respondents’ reactions to the interviews. In addition, given the length and complexity of the interview process, we were not able to systematically assess informant's reactions to individual interview items. Nevertheless, interviewers and informants both had positive overall perceptions of the interviews. The low number of explicit refusals to participate or interruptions of the interviews themselves also indicated a high level of acceptance. Moreover, the relatively small number of respondents per subject may potentially have limited the amount of information that was collected per case. The inclusion of other sources of information (such as medical and criminal records) aimed to minimise this limitation.

Inherent limitations in the psychological autopsy method also apply to our study (64). Some diagnostic criteria may be difficult to be endorsed by respondents since they address inner feelings, which can determine underreporting. Also, the knowledge that someone had a psychiatric history may bias the response to individual items. An overestimation of symptoms may also occur because informants may believe that someone has to be mentally unwell to commit suicide, or as an attempt to construct meaning of the death. Finally, the mental state of the informant was not formally assessed, and it could also play a role in their responses.

In conclusion, indications from this initial evaluation of the study's process and instruments suggest that psychological autopsy is a suitable and valid methodology for suicide studies with Inuit in Nunavut. Additionally informants indicated that they benefited from the psychological autopsy interviews. The method is able to address questions relevant to evidence-based suicide prevention.
